# Investigating a new alarming outbreak of *flavescence dorée* in Tuscany (Central Italy): molecular characterization and *map* gene typing elucidate the complex phytoplasma ecology in the vineyard agroecosystem

**DOI:** 10.3389/fpls.2024.1489790

**Published:** 2024-12-13

**Authors:** Athos Pedrelli, Marco Carli, Alessandra Panattoni, Elisa Pellegrini, Domenico Rizzo, Cristina Nali, Lorenzo Cotrozzi

**Affiliations:** ^1^ Department of Agriculture, Food and Environment, University of Pisa, Pisa, Italy; ^2^ Department of Biological and Environmental Sciences and Technologies, University of Salento, Lecce, Italy; ^3^ Department of Agriculture Technologies, Food, Environment and Forestry, University of Florence, Florence, Italy; ^4^ Regional Phytosanitary Service, Laboratory of Phytopathological Diagnostics and Molecular Biology, Pistoia, Italy

**Keywords:** *Alnus glutinosa*, *Clematis vitalba*, *Dictiophara europaea*, gene typing, grapevine yellows, *Scaphoideus titanus*, *Vitis vinifera*

## Abstract

*Flavescence dorée* (FD) is a major grapevine disease in Europe, despite the quarantine status of its causal agent [FD phytoplasma (FDp)] and the mandatory monitoring and vector control practices. As alarming FD epidemic outbreaks continue to appear in Tuscany (Central Italy), a 4-year survey was carried out in the main wine-growing areas of the region, where FD presence was investigated in both primary and secondary FDp hosts and vectors, i.e., *Vitis vinifera* (VV), *Clematis vitalba* (CV), *Alnus glutinosa* (AG), *Scaphoideus titanus* (ST), and *Dictyophara europaea* (DE). This work i) confirmed FD diffusion in almost the whole of Tuscany and even with an increased occurrence rate (approximately 50% of the samples tested positive); ii) highlighted a complex FDp ecology also in the Tuscan vineyard agroecosystem, as FDp was reported not only in VV and ST but also in secondary vectors (DE) and hosts (AG and CV); iii) reported nine FDp strains (three of which were novel) belonging to all the three methionine aminopeptidase (*map*) clusters, i.e., *map*-FD1, *map*-FD2, and *map*-FD3, with the *map*-FD3/M51 genotype mostly reported in not only VV but also DE and CV; and iv) further confirmed a complex FDp ecology in the vineyard agroecosystem, also by phylogenetic analyses carried out at both Italian and European levels, which also showed some relations between the Tuscan FDp strains and those reported in the Balkan and the French scenarios. We believe that the outcomes reported here will be useful in preventing and controlling the spread of harmful FD.

## 1 Introduction

*Flavescence dorée* (FD) is a major disease of grapevine (*Vitis vinifera*) in Europe, causing both severe yield losses and lower grape quality, with harsh economic consequences in the main wine- and vine-producing countries (EFSA et al., 2020). It is associated with flavescence dorée phytoplasma (FDp), which is transmitted from vine to vine by infected *Scaphoideus titanus* (a monovoltine leafhopper accidentally introduced from North America in the 1950s) through its feeding activity on leaves (Chuche and Thiéry, 2014). FD symptoms develop gradually throughout the summer, reaching their peak appearance in late August–September. Affected grapevines exhibit a drooping appearance due to absent lignification, and leaves generally become crisp, brittle, and curled downward, with white and red grape varieties showing yellowing and reddening, respectively. FDp also leads to inflorescence and berry death (Boulent et al., 2020). FDp-infected plants die or become less productive over the years, also constituting a potential source of inoculum. These symptoms are identical to those of *Bois noir* (BN), another phytoplasma disease associated with ‘*Candidatus* phytoplasma solani’ (BNp); to distinguish FD from BN, both referred to as “grapevine yellows”, laboratory analyses are needed (Chuche and Thiéry, 2014). Despite the quarantine status of FDp (included in the A2 EPPO list; EPPO, 2024a) and the (costly) mandatory monitoring and vector control practices carried out with a high pesticide consumption (there is no phytoplasma control method and no cure for infected plants), FD is still spreading in several European countries (EFSA et al., 2020).

Recent studies have reported that the epidemiological cycle of FDp could be more complex than the vine-to-vine transmission by *S. titanus*, as i) FDp was also found in other plant hosts (mostly asymptomatic) located close to vineyards, such as *Alnus glutinosa* (Angelini et al., 2004), *Clematis vitalba* (Casati et al., 2017), *Ailanthus altissima* (Plavec et al., 2019), and *Salix* spp (Jarausch et al., 2023); ii) *S. titanus* can occasionally feed on other plant species that may act as FDp reservoirs, although the insect cannot complete its development on them (Trivellone et al., 2013), and these host plants may act as pathogen reservoirs (Chuche and Thiéry, 2014; Moussa et al., 2023); and iii) FDp can be transmitted to grapevines by other insects, such as *Allygus* spp., *Dictyophara europaea*, *Orientus ishidae*, and *Phlogotettix cyclops* (Ripamonti et al., 2020; Galetto et al., 2023). Overall, given the abovementioned alarming signs of FDp diffusion in many European viticultural areas and the ecological complexity of FDp, further investigations are needed to ascertain the causes and origins of new outbreaks and predict the route of disease spread, with a precise identification and typing of FDp strains playing a crucial role in this context (Arnaud et al., 2007).

Based on nucleotide sequence analysis of the gene *16S rRNA* and the intergenic spacer between *16S* and *23S rRNA*, FDp has been assigned to the 16SrV taxonomic group (elm yellows group) and specifically to the subgroups 16SrV-C and 16SrV-D (Lee et al., 1998; Angelini et al., 2001; Davis and Dally, 2001; Lee et al., 2004; EPPO, 2024b). However, the variability of *16S rRNA* is not sufficient to account for the abovementioned emerging ecological differences in FDp biological cycles (i.e., secondary vectors and hosts). In contrast, molecular and phylogenetic analyses of the epidemiologically informative genetic loci (i.e., methionine aminopeptidase, *map*; ribosomal proteins, *rp*; preprotein translocase membrane subunit, *secY*; subunit B of the exonuclease family protein, *uvrB-degV*; and adhesion-related proteins, *vmp*) allowed the identification of different FDp genetic clusters. Specifically, the *map* gene was shown to be the most auspicious marker for the genetic characterization of phytoplasma strains within the 16SrV group (Plavec and Music, 2023) and allowed the recognition of three FDp genetic clusters, namely, *map*-FD1 (16SrV-C), *map*-FD2 (16SrV-C and 16SrV-D), and *map*-FD3 (16SrV-C), each of which differs in genetic variability and more importantly is characterized by specific vectors, hosts, and geographical distribution (Arnaud et al., 2007; Malembic-Maher et al., 2020). To date, only *map*-FD1 and *map*-FD2 have been found in France and Switzerland (Arnaud et al., 2007; Casati et al., 2017; Malembic-Maher et al., 2020), only *map*-FD2 has been reported in Portugal (De Sousa et al., 2010), only *map*-FD2 and *map*-FD3 have been identified in Austria and Slovenia (Mehle et al., 2011; Reisenzein and Steffek, 2011), and *map*-FD3 has been exclusively found in Serbia (Malembic-Maher et al., 2020; Krnjajić et al., 2007; Holz et al., 2016; Krstić et al., 2018). All three genotype clusters have been reported in Croatia, Hungary, and Italy (Angelini et al., 2001; Martini et al., 2002; Rossi et al., 2019; Kriston et al., 2019; Plavec et al., 2019; Malembic-Maher et al., 2020).

Although FDp has been present for a long time in Italy (Belli et al., 2010)—beyond the fact that Italy is renowned for consistently ranking as the world’s largest wine producer and for producing some of the world’s most excellent wines (Ohana-Levi and Netzer, 2023)—studies that have focused on FDp diffusion and genetic typing of FDp strains have been mostly performed in regions of Northern Italy (Schubert, 2017; Angelini et al., 2001; Martini et al., 2002; Rossi et al., 2019), leaving out other major viticultural areas such as Tuscany (Central Italy), which is characterized by a warmer Mediterranean climate. The presence of FDp in Tuscany was first reported in a northern province more than two decades ago (Bertaccini et al., 2003) and confirmed more recently in other central and southern traditional viticultural areas by field surveys carried out from 2012 to 2015 (Rizzo et al., 2018) and in 2017 (Pierro et al., 2023), also reporting an increasing diffusion from 2%–11% to 17% of positive samples, respectively. However, only very few FDp strains from Tuscany have been characterized molecularly, mainly resulting in cluster *map*-FD1 strains (Arnaud et al., 2007; Malembic-Maher et al., 2011; Malembic-Maher et al., 2020). Actually, we carried out the first multi-locus sequence typing (MLST) of 15 FDp strains isolated in 2017, determined through the analysis of the *16S rRNA*, *rp*, and *secY* genes, showing that i) the Tuscan FDp strains are within the subgroup 16SrV-C, with many strains resulting very close to others previously identified not only in Northern Italy but also in France, Slovenia, and Serbia; and ii) the collective genotype (16S rRNA/*rp*/*secY*) of the Tuscan FDp strains is unique and constitutes a new highly homogeneous lineage (Pierro et al., 2023). However, comprehensive of the population and molecular features of the Tuscan FDp strains by *map* gene typing (i.e., the most suitable approach for the genetic characterization of FDp, as stated above; Plavec and Music, 2023) has not been performed. Furthermore, a study of FDp ecology in the Tuscan vineyard agroecosystem (i.e., characterizing FDp strains collected also from secondary hosts and vectors) has never been carried out before the present study.

To address these knowledge gaps, and as alarming FD epidemic outbreaks continue to appear in Tuscany (as reported by the monitoring activity carried out by both the Department of Agriculture, Food and Environment of the University of Pisa, and the Regional Phytosanitary Service of the Region of Tuscany), also emerging in diverse habitats, this study aimed to i) elucidate the current FDp diffusion through a long-term field survey (2020–2023) carried out in the major viticultural areas of Tuscany, ii) investigate the FDp epidemiological cycle in the whole vineyard agroecosystem, iii) determine the genetic diversity among the collected FDp strains by *map* gene typing, and iv) elucidate the evolutionary relationships of Tuscan FDp strains with those reported in Italy, as well as in Europe. We anticipate that the outcomes reported here will be useful in preventing and controlling the spread of harmful FD.

## 2 Materials and methods

### 2.1 Field surveys and sampling of leaf and insect

**Figure 1:**
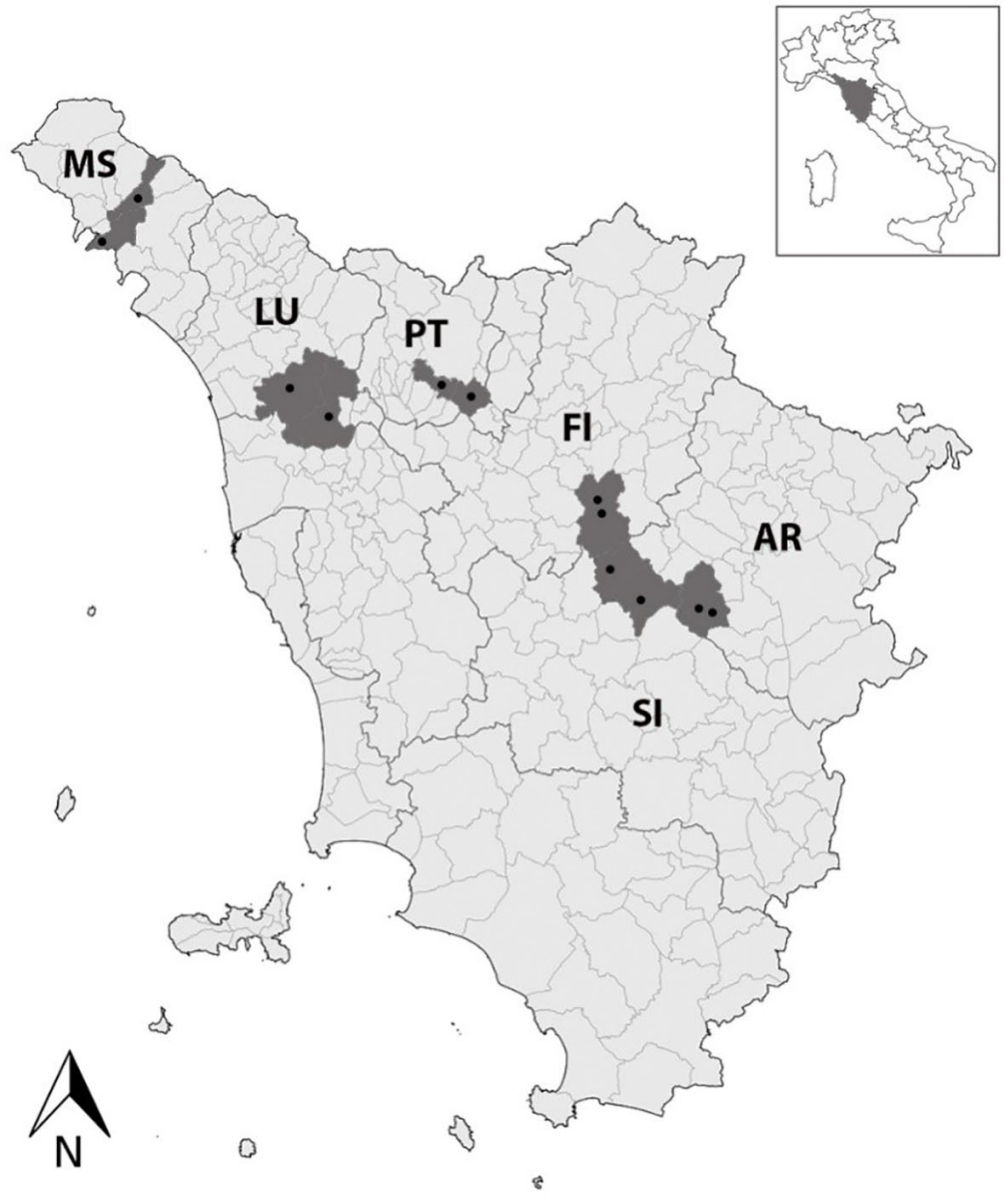
A map of Tuscany (Central Italy) showing the 12 investigated vineyards (black dots) located in the main wine-growing areas, i.e., Aulla and Licciana Nardi (Massa, MS), Capannori and Aquilea (Lucca, LU), Serravalle Pistoiese and Quarrata (Pistoia, PT), Gaiole in Chianti, Radda in Chianti (Siena, SI), Greve in Chianti, San Casciano in Val di Pesa (Florence, FI), and Bucine and Montevarchi (Arezzo, AR).

Experimental activities were carried out from 2020 to 2023. In total, 12 vineyards located in the main wine-growing areas of Tuscany (Central Italy; two vineyards per province), i.e., Aulla and Licciana Nardi (Massa, MS), Capannori and Aquilea (Lucca, LU), Serravalle Pistoiese and Quarrata (Pistoia, PT), Gaiole in Chianti, Radda in Chianti (Siena, SI), Greve in Chianti, San Casciano in Val di Pesa (Florence, FI), and Bucine and Montevarchi (Arezzo, AR) were selected in collaboration with the Regional Phytosanitary Service of the Region of Tuscany (**Figure 1**). Leaf samples were collected at the end of August/September from *V. vinifera* cv. Sangiovese (VV), as well as from *C. vitalba* (CV) and *A. glutinosa* (AG) located at the edges of the examined vineyards. Concomitantly, *S. titanus* (ST) and *D. europaea* (DE) were collected using chromotropic traps placed in the vineyards at grapevine canopy height, as well as in the nearby wood margins. A total of 276 samples were collected, i.e., 218 VV, 24 ST, 20 DE, 10 CV, and 4 AG. The leaves were placed in coolers and quickly carried to the Plant Pathology Laboratory of the Department of Agriculture, Food, and Environment (DAFE) of the University of Pisa, where their midribs were dissected and stored at −20°C. The insects were recovered from traps, identified by experts of the Entomology Laboratory of DAFE, placed in 70% (v/v) ethanol, and stored at +4°C.

### 2.2 DNA isolation

A cetyltrimethylammonium bromide (CTAB) buffer procedure was used to extract DNA from leaves, according to Li et al. (2008), with minor modifications (Pedrelli et al., 2023a; Pedrelli et al., 2023c). Approximately 0.5 g of leaf midribs was powdered in liquid nitrogen, added to 5 mL of 2% (w/v) CTAB buffer, and incubated at +65°C for 15 min. DNA was extracted by one volume of chloroform:isoamyl alcohol (24:1, v/v) and precipitated with one volume of isopropanol. The pellets were then washed with 70% (v/v) ethanol, air-dried, and dissolved in 80 μL of RNase/DNase-free water.

In contrast, DNA from insects was extracted following Marzachi et al. (1998), with minor modifications. Every single insect was inserted into a 2.0-mL screw cap microcentrifuge tube containing six 2.8-mm ceramic (zirconium oxide) slingshot beads (Bertin Technologies SAS, Montigny-le-Bretonneux, France), and 400 μL of 2.5% (v/v) CTAB buffer was added. Then, tubes were centrifuged at 7,200 *g* for 10 s using Precellys Evolution Touch (Bertin Technologies SAS). The formed suspensions were incubated at +60°C for 30 min and then extracted with chloroform:isoamyl alcohol (24:1, v/v). DNA was precipitated by adding one volume of cold isopropanol and recovered by centrifugation at 14,000 *g* for 3 min, washed with 70% (v/v) ethanol, air-dried, and re-suspended in 80 μL of RNase/DNase-free water.

The DNA (extracted from both leaves and insects) was finally purified using the DNeasy PowerClean Pro Cleanup Kit (Qiagen, Venlo, The Netherlands) according to the manufacturer’s instructions, checked for their quality and quantity using a QIAExpert system (Qiagen), and stored at −80°C until molecular analysis.

### 2.3 16S rRNA phytoplasma subgroup V and XII detection and *map* gene amplification

Samples were tested for the presence of *16S rRNA* phytoplasma subgroups V (16SrV) and XII (16SrXII) by real-time polymerase chain reaction (qPCR) using specific primer pairs (Angelini et al., 2007). The qPCR assays were carried out by a Rotor-Gene Q Thermocycler (Qiagen) using a 20-μL reaction volume containing a QuantiNova Probe (Qiagen). Moreover, phytoplasma identification was confirmed by end-point PCR for *16S rRNA* using the universal P1/P7 primer pair (Deng and Hiruki, 1991), followed by the R16(V)F1/R1 and R16(XII)F1/R1 ones for the detection of 16SrV and 16SrXII, respectively (Lee, 1994). Differently, the protocol described by Arnaud et al. (2007) was adopted to amplify the *map* genetic loci. PCRs were conducted employing the FD9F5/MapR1 primer pair (direct PCR), followed by the FD9F6/MapR2 one (nested PCR). PCR and nested PCR were performed using a C1000 Touch^®^ thermal cycler (Bio-Rad, Hercules, CA, USA) in reaction volumes of 25 μL and 50 μL, respectively, including DreamTaq PCR Master Mix and DreamTaq Green PCR Master Mix (Thermo Fisher Scientific, Waltham, MA, USA), respectively. PCR-amplified products were observed by electrophoresis through 1.2% (w/v) agarose gel and sequenced following the Sanger DNA method (Eurofins, Ebersberg, Germany). Positive, healthy, and no-template controls, were included in each molecular assay.

### 2.4 16S rRNA and *map* gene amplicon sequencing, characterization, and *in silico* analysis

Amplicons from 16SrV-positive samples and the corresponding *map* gene were sequenced following the Sanger DNA method by the Eurofins Genomics commercial service (Ebersberg, Germany). Group/subgroup assignment (16SrV-C/D; 16SrXII) of the 16S rRNA nucleotide sequences was accomplished using the online tool iPhyClassifier (Zhao et al., 2009). *map* gene amplicons were typed by restriction fragment length polymorphism (RFLP) analysis performed through the *Alu*I and *Eco*72I restriction enzymes in order to characterize their *map* cluster (i.e., *map*-FD1, *map*-FD2, and *map*-FD3; Arnaud et al., 2007). Nucleotide and amino acid sequences were analyzed *in silico* using the BioEdit software (Hall, 1999), and nucleotide sequences were also compared in BLASTn (www.ncbi.nlm.ni.gov) and to the dataset provided by Malembic-Maher et al. (2020). Alignments were performed to observe the presence of non-synonymous (dN) and synonymous (dS) single-nucleotide and amino acid polymorphisms, and a sequence identity determination was carried out by the sequence identity matrix application of BioEdit. The role of natural selection in the FDp population using the Codon Z-test by the Nei–Gojobori method and the dN/dS ratio by the Felsenstein (1981) model was estimated using MEGA X (Pedrelli et al., 2023b). Phylogenetic analyses were carried out in MEGA X using the maximum likelihood (ML) and neighbor-joining methods (using the Jukes–Cantor and p-distance models, respectively) with 1,000 bootstrap replicates (Kumar et al., 2018). A BN isolate (AM990988) was used as an outgroup. The used *map* strain dataset is reported in **Supplementary Table S1**.

## 3 Results and discussion

### 3.1 Diffusion of FD was confirmed in almost the whole Tuscany and even with an increased occurrence rate

The present study first confirmed that, despite the regulation that requires FDp monitoring and vector control practices, along with the destruction of vineyards when they are infected with FDp, this threatening disease is still present in Tuscany (Central Italy; Bertaccini et al., 2003; Rizzo et al., 2018; Pierro et al., 2023), in the regions of Northern Italy (Rigamonti et al., 2023; Quaglino et al., 2024), and other European areas (e.g., Plavec et al., 2019; Krstić et al., 2022). The typical FD leaf symptoms, i.e., reddening and downward rolling, observed on all collected VV leaves were indeed confirmed by molecular assays, which reported the presence of FDp in a large number of samples (47%; **Table 1**), whereas only four VV samples tested positive for BNp (2%), in accordance with Pierro et al. (2018), and with no mixed infection. FDp-positive samples were collected in all the investigated Tuscan provinces (i.e., Arezzo, Florence, Lucca, Massa, Pistoia, and Siena; **Figure 1**). These outcomes confirm that the FD outbreak has extended to almost the whole of Tuscany (Pierro et al., 2023), but also suggest an alarming increase in the disease occurrence rate, given the rise in the percentage of FDp-positive samples from 2%–11% and 17% reported in previous surveys (Rizzo et al., 2018; Pierro et al., 2023) to almost 50% of the present study.

**Table 1:** Host (VV, *Vitis vinifera*; CV, *Clematis vitalba*; AG, *Alnus glutinosa*; ST, *Scaphoideus titanus*; DE, *Dictyophara europaea*) and samples tested positive for flavescence dorée phytoplasma (FDp)/samples investigated by PCR assay; subgroup obtained by iPhyClassifier of FDp strains identified by *16s rRNA* typing; and name, frequency of occurrence, cluster, associated genotype, and accession number and related similarity obtained by BLASTn analysis (AM238512, isolate FD70; CP097583, isolate CH; LT222008, isolate AI-AL4; OQ185203, isolate 6023/2021) of FDp strains identified by *map* typing.

Host	FDp	*16S rRNA*	*map*				
		Subgroup	Name	Frequency (%)	Cluster	Genotype	Accession number (similarity, %)
VV	103/218	FDV-C	ViTos997	2	FD2	M54	CP097583 (100.00)
			ViTos601	62	FD3	M51	OQ185203 (99.85)
			ViTos15	36	FD1	M50	AM238512 (100.00)
CV	6/10	FDV-C	CleTos8	100	FD3	M51	OQ185203 (100.00)
AG	4/4	FDV-C	AlnTos8	75	FD1	M113	LT222008 (100.00)
			AlnTos9	25	FD2	M54	CP097583 (99.61)
ST	8/24	FDV-C	ScaTos29	100	FD1	M50	AM238512 (100.00)
DE	6/20	FDV-C	DicTos27	50	FD3	M51	OQ185203 (99.85)
			DicTos20	50	FD3	M51	OQ185203 (100.00)

### 3.2 FDp was also reported in secondary vectors and hosts, highlighting a complex FDp ecology in Tuscany

A second major achievement of the present study was the first detection of FDp in multiple plants and insects collected inside and outside vineyards in Tuscany, although the presence in vineyard agroecosystems of different leafhopper and plant species infected with FDp was already reported in other regions of Northern Italy (Ripamonti et al., 2021; Rigamonti et al., 2023; Quaglino et al., 2024) and Europe (Rizzoli et al., 2021; Radonjić et al., 2023; Kogej Zwitter et al., 2023; Oggier et al., 2023). Although no macroscopic symptoms were reported on the collected CV and AG leaves, the molecular assays reported the presence of FDp in 60% of CV samples and all AG ones, and interestingly, FDp was also reported in 33% and 30% of ST and DE samples, respectively (**Table 1**). It is, therefore, reasonable to hypothesize that in Tuscany, the ecological cycle of FDp may be related exclusively not only to the grapevine-specific feeding of *S. titanus*, but also to that of other insect vectors and plant hosts (Casati et al., 2017). Previous studies have suggested that the vector competence of FDp transmission could be widespread and proposed the question of whether in a viticultural area the range/number of effective insect vector species could be determined by the availability of suitable host plant species in a system where FDp strains flow among many different hosts, inside and outside the vineyards (Bressan et al., 2006).

### 3.3 The *map* gene typing of nine FDp strains collected in Tuscany showed that they belong to all three *map* clusters, with the *map*-FD3/M51 genotype mostly reported not only in VV but also in DE and CV

The abovementioned diagnostic results were confirmed by end-point PCRs targeting the *16S rRNA* gene, which also allowed us to obtain amplicons of the expected size. Specific primers for the *map* genetic loci enabled the generation of a comparable number of amplicons to those obtained from samples that previously tested positive for FDp. As expected, the iPhyClassifier, utilized using our *16S rRNA* sequences as queries, revealed that the identified FDp strains belonged to the 16SrV-C subgroup (**Table 1**), not only those found in VV samples, which is in agreement with previous observations in Tuscany (Pierro et al., 2023), but also those reported in the other primary and secondary FDp insect vectors and plant hosts.

The alignment of the 127 *map* nucleotide sequences (657–763 bp) and their RFLP analysis allowed the identification of nine FDp strains, which (as expected) belonged to all the known three *map* clusters (Arnaud et al., 2007; Malembic-Maher et al., 2020; Plavec and Music, 2023): four to *map*-FD3, three to *map*-FD1, and two to *map*-FD2 (**Table 1**). Actually, the identification of two of our FDp strains belonging to the *map*-FD2 cluster disagrees with the association of all the *16S rRNA* sequences to the 16SrV-C subgroup reported above (Arnaud et al., 2007; Zhao et al., 2009). However, the occurrence of these FDp strains changed depending on the type of samples collected in the Tuscan vineyard agroecosystem: genotypes collected in VV mostly belonged to the *map*-FD3 cluster (62% of FDp-positive samples), followed by *map*-FD1 (36%) and *map*-FD2 (2%), whereas CV was infected by only one genotype belonging to *map*-FD3 (100%), and the most frequent cluster in AG was *map*-FD1 (75%), followed by *map*-FD2 (25%). All FDp strains collected from ST and DE belonged to the *map*-FD1 and *map*-FD3 clusters, respectively.

BLASTn analysis of the nine *map* nucleotide sequences allowed the identification of three novel FDp strains each collected in VV, DE, and AG, and named ViTos601, DicTos27, and AlnTos9, respectively (**Table 1**). ViTos601 and DicTos27 showed a 99.85% similarity with isolate 6023/2021 (OQ185203) previously found in VV in the Czech Republic and were related to the M51 genotype; AlnTos9 was characterized by a 99.61% similarity with isolate CH (CP097583) previously found in an unidentified insect in Switzerland and was associated with the M54 genotype (Malembic-Maher et al., 2020). For the remaining six isolates, two were collected from VV (ViTos997 and ViTos15) and one each from CV, AG, ST, and DE (CleTos8, AlnTos8, ScaTos29, and DicTos20, respectively). All of them were identical (100.00% similarity) to previously identified isolates: ViTos997 to the abovementioned CH (CP097583, M54 genotype), ViTos15 and ScaTos29 to FD70 (AM238512, M50 genotype, previously reported in *Catharanthus roseus* in France), CleTos8 and DicTos20 to the abovementioned 6023/2021 (OQ185203, M51 genotype), and AlnTos8 to AI-AL4 (LT222008, M113 genotype, previously reported in AG in Tuscany); Malembic-Maher et al., 2020; **Table 1**). All the identified isolates were deposited in GenBank (www.ncbi.nlm.ni.gov; **Supplementary Table S1**).

Contrary to previous studies reporting the *map*-FD2/M54 FDp strain as the most prevalent genotype in Northern Italy (Rigamonti et al., 2023), here, *map*-FD3/M51 (ViTos601) was the most prevalent genotype reported in VV, followed by *map*-FD1/M50 (ViTos15), and only a few VV samples were infected with *map*-FD2/M54 (ViTos997). In fact, the *map*-FD1/M50 genotype (ViTos15) was also the only one reported in ST (ScaTos29), confirming the role of this insect as the main FDp vector in vineyards (Rizzoli et al., 2023). However, the fact that the novel *map*-FD3/M51 genotype, largely reported in VV (ViTos601) was also found in DE (DicTos27) hypothetically suggests that DE is also capable of (occasionally) transmitting FDp directly to VV (Radonjić et al., 2023). Furthermore, the association of both FDp strains reported in DE and (asymptomatic) CV (DicTos20 and CleTos8) with the same *map*-FD3/M51 genotype confirms the ability of FDp transmission between these secondary insect vectors and plant hosts, according to previous studies reporting the same genotype in other Italian and European regions (Angelini et al., 2004; Filippin et al., 2009; Linhartová et al., 2023; Quaglino et al., 2024). This prevalence of the *map*-FD3/M51 genotype in secondary FDp host plants and insect vectors also confirms a link between Tuscany and Eastern European nations like Croatia, Montenegro, and Serbia (Plavec et al., 2019; Krstić et al., 2022; Radonjić et al., 2023). Finally, AG was confirmed to be a potential (asymptomatic) reservoir of FDp, here, with the *map*-FD1/M113 and *map*-FD2/M54 related genotypes, confirming its capacity to host multiple FDp genotypes (Malembic-Maher et al., 2020). Numerous investigations have demonstrated the ability of insect vectors such as *Allygus* spp. and *Oncopsis* spp. to acquire FDp from AG and transfer it to other hosts, even if VV is not currently included (Maixner et al., 2000; Jarausch et al., 2021).

### 3.4 The phylogenetic analysis carried out at the Italian and European levels confirmed a complex FDp ecology, with some relations with the Balkan and the French scenarios

Identity levels between *map* nucleotide sequences ranged from 97.84% to 100.00%, with the maximum difference observed between ViTos997 and AlnTos8 and the minimum ones between ViTos601 and DicTos27, ViTos15, and ScaTos9, and CleTos8 and DicTos20 (**Table 2**; the 100.00% similarities are reported above). Overall, the genetic variability (π) was estimated to be 0.013 ± 0.003 (mean ± standard error) if using only the FDp strains reported in the present study and 0.010 ± 0.003 if including also the other FDp strains previously collected in Tuscany. These values are in agreement with those emerging from the analysis of FDp strains found in Veneto (n = 16, 0.017 ± 0.003) and Friuli Venezia Giulia (n = 6, 0.015 ± 0.003; other Italian regions were not taken into account because of the low number of FDp strains available). The amount of SNPs among the nine sequences of the FDp strains reported here was 54: 13 nt for ViTos997 with 3 dN, 11 nt for AlnTos9 with 4 dN, 10 nt each for ScaTos29 and ViTos15 with 3 dN each, 8 nt for AlnTos8 with 2 dN, and 1 nt each for CleTos8 and DicTos20 without dN. The overall dN/dS ratio was 0.08 (*p* = 0.001), taking into account only the FDp strains collected in the present study, 0.10 (*p* = 0.002), including also those previously reported in Tuscany, and 0.18 and 0.19, analyzing those from Veneto (*p* < 0.001) and Friuli Venezia Giulia (*p* = 0.001), respectively. These outcomes suggest a satisfactory adaptation of FDp in Tuscany, even more than in the other investigated Italian regions, a phenomenon that likely favors a high stability of the FDp genetic structure (Escriu, 2017; Pedrelli et al., 2024).

**Table 2:** Identity matrix between flavescence dorée phytoplasma (FDp) nucleotide (lower left) and deduced amino acid (upper right) sequences recovered in Tuscan vineyards located in the wine-growing areas.

Isolate	ViTos601	ViTos15	ViTos997	CleTos8	AlnTos8	AlnTos9	ScaTos29	DicTos20	DicTos27
ViTos601		99.47	98.94	100.00	98.94	98.94	99.47	100.00	100.00
ViTos15	98.57		98.40	99.47	99.47	98.40	100.00	99.47	99.47
ViTos997	98.02	98.02		98.94	97.87	100.00	98.40	98.94	98.94
CleTos8	99.82	98.39	98.21		98.94	98.94	99.47	100.00	100.00
AlnTos8	98.57	99.64	97.84	98.39		97.87	99.47	98.94	98.94
AlnTos9	98.39	98.21	99.64	98.57	98.21		98.40	98.94	98.94
ScaTos29	98.57	100.00	98.02	98.39	99.64	98.21		99.47	99.47
DicTos20	99.82	98.39	98.21	100.00	98.39	98.57	98.39		100.00
DicTos27	100.00	98.57	98.02	99.82	98.57	98.39	98.57	99.82	

**Figure 2:**
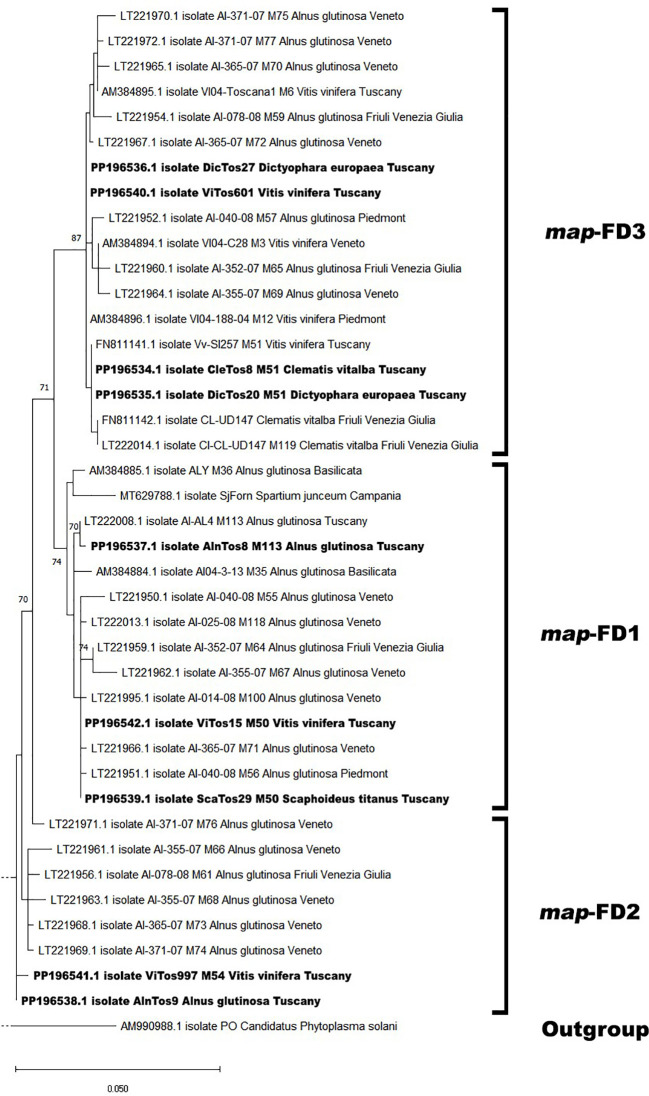
Phylogenetic tree of flavescence dorée phytoplasma (FDp) isolates from Italy reconstructed from partial *map* genes. The tree was generated by maximum likelihood (ML) method using the Jukes–Cantor model of evolution for nucleotides. The significance of each branch was evaluated by constructing 1,000 trees in bootstrap analysis. Bootstrap values >70 are shown. The scale represents a distance of 0.050 substitutions per site. The isolates sequenced in this study are in bold, and the subdivisions between clusters are reported on the right. The Bois noir phytoplasma (BNp) isolate (AM990988) was used as an outgroup. A more detailed version of the phylogenetic tree is reported in S**upplementary Figure S1**.

**Figure 3:**
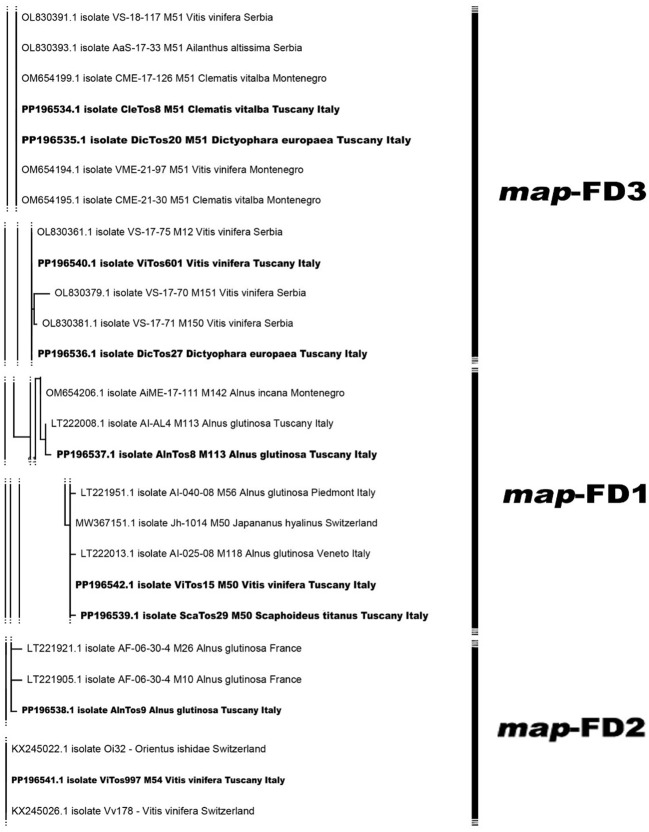
Phylogenetic tree of flavescence dorée phytoplasma (FDp) isolates from Europe reconstructed from partial *map* genes. The tree was generated by maximum likelihood (ML) method using the Jukes–Cantor model of evolution for nucleotides. The significance of each branch was evaluated by constructing 1,000 trees in bootstrap analysis. Bootstrap values >70 are shown. The scale represents a distance of 0.050 substitutions per site. The isolates sequenced in this study are in bold, and the subdivisions between clusters are reported on the right. The Bois noir phytoplasma (BNp) isolate (AM990988) was used as an outgroup. A more detailed version of the phylogenetic tree is reported in **Supplementary Figure S2**.

The phylogenetic analysis conducted using the available Italian *map* nucleotide sequences (i.e., those reported here and previously submitted to NCBI; **Supplementary Table S1**) confirmed the occurrence of the three *map* clusters (Arnaud et al., 2007) with high bootstrap values (**Figure 2**; **Supplementary Figure S1**), with the nine FDp strains reported in the present study distributed among them. In the *map*-FD3 cluster, DicTos27 and ViTos601 (related to the M51 genotype) were close together and close to isolates previously reported in AG in the Northern Regions, i.e., Veneto (AI-365-07, LT221967, M72) and Piedmont (AI-040-08, LT221952, M57). Conversely, DicTos20 and CleTos8 (M51) were closely related to the isolate Vv-SI257 (FN811141, M51) found in VV in Tuscany, and interestingly, they also grouped with isolates CL-UD147 (FN811142) and CI-CL-UD147 (LT222014, M119) collected in CV in Friuli Venezia Giulia, i.e., the only isolates reported in CV at the Italian level. In fact, using all the available European *map* nucleotide sequences, DicTos27 and ViTos601, as along with DicTos20 and CleTos8, were also found to be related to other FDp strains collected in the Balkans (i.e., Montenegro and Serbia): the former was close to strains reported only in VV, and the latter was close to strains also found in CV (and *A. altissima*, an invasive alien species; Pisuttu et al., 2020, 2023; **Figure 3**; **Supplementary Figure S2**). These results confirm the relationship between DE and CV and highlight that CV is (so far) exclusively infected by *map*-FD3 genotypes also in Tuscany (Malembic-Maher et al., 2020; Radonjić et al., 2023).

In contrast, the *map*-FD1 cluster was found to be mostly composed of isolates collected only in AG in Northern Regions and in the other secondary FDp host *Spartium junceum* in Campania (MT629788), and, indeed, included the FDp strain reported here, AlnTos8 (M113 genotype), which showed a high bootstrap value with isolate AI-AL4 (LT222008, M113) already reported in AG in Tuscany (**Figure 2**; **Supplementary Figure S1**). Accordingly, the phylogenetic analysis conducted at the European level also placed AlnTos8 close to a strain previously collected in *Alnus incana*, again in the Balkans (Montenegro; **Figure 3**). Interestingly, however, we also reported the occurrence in the *map*-FD1 cluster of FDp strains reported in VV and ST (ViTos15 and ScaTos29, M50 genotypes), which were also placed close to the isolates AI-40-08 (LT221951, M56), AI-365-07 (LT22966, M71), and AI-014-08 (LT221995, M100) found in AG in Piedmont and Veneto (**Figure 2**; **Supplementary Figure S1**). This first Italian submission of *map*-FD1/M50 genotypes collected in VV and ST confirms the complexity of the FDp ecology in the vineyard ecosystem also in Tuscany (Casati et al., 2017) and proposes a scenario close to the French one (Malembic-Maher et al., 2020; Rigamonti et al., 2023), as further confirmed by the close relation found between ViTos15 and ScaTos29 to FDp strains isolated from VV in France, highlighted by the European phylogenetic analysis (**Figure 3**; **Supplementary Figure S2**). Similarly, in the *map*-FD2 cluster characterized only by FDp strains collected in AG, we reported not only a novel FDp strain found in AG (AlnTos9, M54 related genotype; **Figure 2**; **Supplementary Figure S1**), which was also close to other FDp strains reported in AG in Europe (**Figure 3**; **Supplementary Figure S2**) but also an FDp strain collected in VV (ViTos997, M54; **Figure 2**). This interesting occurrence of a *map*-FD2/M54 genotype in VV was previously reported (Rigamonti et al., 2023) and was confirmed by the very close relation between ViTos997 and FDp strains previously collected in VV in Switzerland, as highlighted by the phylogenetic analysis conducted at the European level (**Figure 3**; **Supplementary Figure S2**). These outcomes further confirm the complexity of the FDp ecology, although the way in which the two hosts VV and AG interact in vineyard agroecosystems is still not clear, likely due to the lack of observations in secondary insect vectors.

Finally, the *map-deduced* amino acid sequences revealed an identity ranging from 97.87%, reported for AlnTos8 with ViTos997 and AlnTos9, to 100.00% for the others (**Table 2**), and since we obtained a longer portion of the *map* gene, a better evaluation of the amino acid variations was operable. Specifically, the alignment of the amino acid sequences revealed specific patterns for the isolates belonging to the different *map* clusters: F_22_, E_26_, M_42_, G_114_, F_192_, and N209 for *map*-FD3 (ViTos601, DicTos20, DicTos27, and CleTos8); L_22_, E_26_, M_42_, D_114_, V_192_, and T_209_ for *map*-FD2 (ViTos997 and AlnTos9); and F_22_, E_26_, I_42_, G_114_, V_192_, and T_209_ for *map*-FD1 (ViTos15 and ScaTos9), although AlnTos8, which also belongs to *map*-FD1, showed unique shorter F_22_, Q_26_, I_42_, and G_114_ patterns (**Table 3**). These outcomes confirm the potential power of elucidating the amino acid variations to better characterize FDp strains, an approach that has been little used for this phytoplasma (Quaglino et al., 2010), in contrast to that for other plant pathogens (e.g., Pedrelli et al., 2024).

**Table 3:** Single-nucleotide polymorphisms (SNPs) and amino acid changes reported in the FDp strains identified by *map* typing (*map* cluster and associated genotype are reported).

FDp strain	*Map* cluster	Genotype	SNP nucleotide position^a^/amino acid position					
			66/22	76/26	144/42	341/114	574/192	626/209
ViTos601	FD3	M51*	c/F	g/E	g/M	g/G	t/F	a/N
DicTos20	FD3	M51	c/F	g/E	g/M	g/G	t/F	a/N
DicTos27	FD3	M51*	c/F	g/E	g/M	g/G	t/F	a/N
CleTos8	FD3	M51	c/F	g/E	g/M	g/G	t/F	a/N
ViTos997	FD2	M54	a/L	g/E	g/M	a/D	g/V	c/T
AlnTos9	FD2	M54*	a/L	g/E	g/M	a/D	g/V	c/T
ViTos15	FD1	M50	c/F	g/E	t/I	g/G	g/V	c/T
ScaTos29	FD1	M50	c/F	g/E	t/I	g/G	g/V	c/T
AlnTos8	FD1	M113	c/F	c/Q	t/I	g/G	t/F	a/N

Nucleotide (lowercase) and corresponding amino acid (uppercase) variations are reported for each position.

a Bases from the start codon (ATG) of the *map* gene.

*Related genotype.

## 4 Conclusions

The present study presents the first comprehensive determination of the population and molecular features of the Tuscan FDp strains by *map* gene typing, as well as of FDp ecology in the Tuscan vineyard agroecosystem. Based on a 4-year survey carried out in the main wine-growing areas of Tuscany, where FD presence was investigated in both primary and secondary FDp hosts and vectors, this work i) confirmed FD diffusion in almost the whole of Tuscany and even with an increased occurrence rate (approximately 50% of samples tested positive); ii) highlighted a complex FDp ecology also in the Tuscan vineyard agroecosystem, as FDp was reported not only in VV and ST, but also in secondary vectors (DE) and hosts (AG and CV); iii) reported nine FDp strains (three of which were novel) belonging to all the three *map* clusters, i.e., *map*-FD1, *map*-FD2, and *map*-FD3, with the *map*-FD3/M51 genotype mostly reported in not only VV but also DE and CV; and iv) further confirmed a complex FDp ecology also by phylogenetic analyses carried out at both Italian and European levels, which also showed some relations between the Tuscan FDp strains and those reported in the Balkan and French scenarios. We believe that the outcomes reported here will be useful to prevent and control the spread of the harmful FD in Tuscany, and in areas of the world where FD epidemic outbreaks continue to occur.

## Data Availability

The datasets presented in this study can be found in online repositories. The names of the repository/repositories and accession number(s) can be found in the article/**Supplementary Material**.
