# Supplementary material for: Investigating a new alarming outbreak of flavescence dorée in Tuscany (Central Italy): molecular characterization and map gene typing elucidate the complex phytoplasma ecology in the vineyard agroecosystem
Source: Front Plant Sci. 2024 Dec 13;15:1489790. doi: 10.3389/fpls.2024.1489790 (PMC11681383; doi:10.3389/fpls.2024.1489790)
Supplement: Supplementary file 1 [file DataSheet1.pdf]

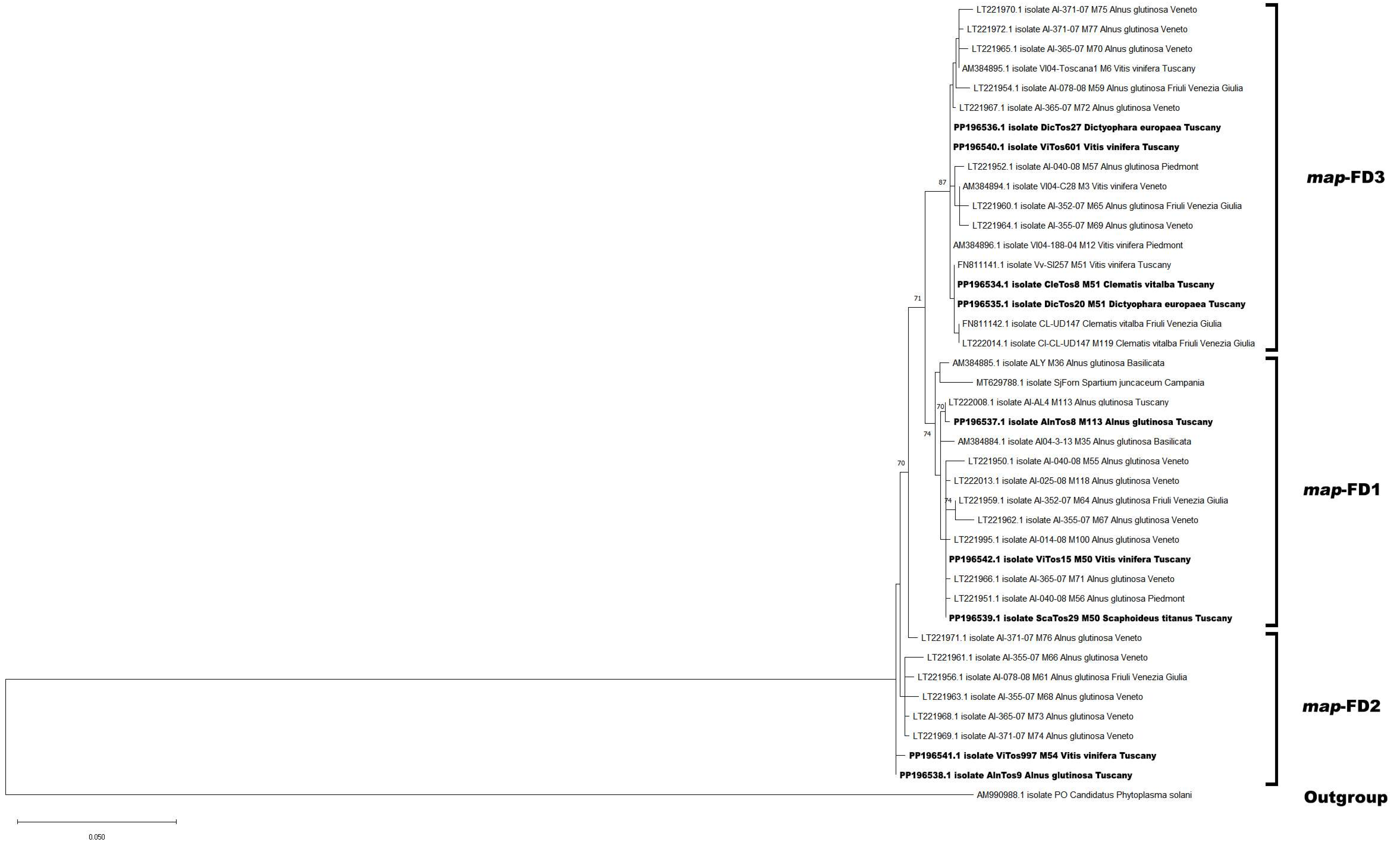

Supplementary Figure S1. Phylogenetic tree of flavescence doreé phytoplasma (FDp) isolates from Italy reconstructed from partial map gene. The tree was generated by Maximum Likelihood (ML) method, using the Jukes-Cantor model of evolution for nucleotide. The significance of each branch was evaluated by constructing 1,000 trees in bootstrap analysis. Bootstrap values > 70 are shown. The scale represents a distance of 0.050 substitutions per site. The isolates sequenced in this study are in bold and the subdivisions between clusters are reported on the right. Bois noir phytoplasma (BNp) isolate (AM990988) was used as outgroup.
