# Supplementary material for: Investigating a new alarming outbreak of flavescence dorée in Tuscany (Central Italy): molecular characterization and map gene typing elucidate the complex phytoplasma ecology in the vineyard agroecosystem
Source: Front Plant Sci. 2024 Dec 13;15:1489790. doi: 10.3389/fpls.2024.1489790 (PMC11681383; doi:10.3389/fpls.2024.1489790)
Supplement: Supplementary file 3 [file Table1.docx]

**Table S1.** Falvescence dorée phytoplasma (FDp) isolates characterized by *map* gene employed in the Italian phylogenetic analysis.

| **Accession number** | **Pathogen** | **Isolate** | ***map* cluster/genotype** | **Host** | **Region** |
| --- | --- | --- | --- | --- | --- |
| AM384884.1 | FDp | AI04-3-13 | FD1/M35 | *Alnus glutinosa* | Basilicata |
| AM384885.1 | FDp | ALY | FD1/M36 | *Alnus glutinosa* | Basilicata |
| AM384894.1 | FDp | VI04-C28 | FD3/M3 | *Vitis vinifera* | Veneto |
| AM384895.1 | FDp | VI04-Toscana1 | FD1/M6 | *Vitis vinifera* | Tuscany |
| AM384896.1 | FDp | VI04-188-04 | FD3/M12 | *Vitis vinifera* | Piedmont |
| FN811141.1 | FDp | Vv-SI257 | FD3/M51 | *Vitis vinifera* | Tuscany |
| LT221950.1 | FDp | AI-040-08 | FD1/M55 | *Alnus glutinosa* | Veneto |
| LT221951.1 | FDp | AI-040-08 | FD1/M56 | *Alnus glutinosa* | Piedmont |
| LT221952.1 | FDp | AI-040-08 | FD3/M57 | *Alnus glutinosa* | Piedmont |
| LT221954.1 | FDp | AI-078-08 | FD3/M59 | *Alnus glutinosa* | Friuli Venezia Giulia |
| LT221956.1 | FDp | AI-078-08 | FD2/M61 | *Alnus glutinosa* | Friuli Venezia Giulia |
| LT221959.1 | FDp | AI-352-07 | FD1/M64 | *Alnus glutinosa* | Friuli Venezia Giulia |
| LT221960.1 | FDp | AI-352-07 | FD3/M65 | *Alnus glutinosa* | Friuli Venezia Giulia |
| LT221961.1 | FDp | AI-355-07 | FD2/M66 | *Alnus glutinosa* | Veneto |
| LT221962.1 | FDp | AI-355-07 | FD1/M67 | *Alnus glutinosa* | Veneto |
| LT221963.1 | FDp | AI-355-07 | FD2/M68 | *Alnus glutinosa* | Veneto |
| LT221964.1 | FDp | AI-355-07 | FD3/M69 | *Alnus glutinosa* | Veneto |
| LT221965.1 | FDp | AI-365-07 | FD3/M70 | *Alnus glutinosa* | Veneto |
| LT221966.1 | FDp | AI-365-07 | FD1/M71 | *Alnus glutinosa* | Veneto |
| LT221967.1 | FDp | AI-365-07 | FD3/M72 | *Alnus glutinosa* | Veneto |
| LT221968.1 | FDp | AI-365-07 | FD2/M73 | *Alnus glutinosa* | Veneto |
| LT221969.1 | FDp | AI-371-07 | FD2/M74 | *Alnus glutinosa* | Veneto |
| LT221970.1 | FDp | AI-371-07 | FD3/M75 | *Alnus glutinosa* | Veneto |
| LT221971.1 | FDp | AI-371-07 | FD2/M76 | *Alnus glutinosa* | Veneto |
| LT221972.1 | FDp | AI-371-07 | FD3/M77 | *Alnus glutinosa* | Veneto |
| LT221995.1 | FDp | AI-014-08 | FD3/M100 | *Alnus glutinosa* | Veneto |
| LT222008.1 | FDp | AI-AL4 | FD1/M113 | *Alnus glutinosa* | Tuscany |
| LT222013.1 | FDp | AI-025-08 | FD1/M118 | *Alnus glutinosa* | Veneto |
| LT222014.1 | FDp | CI-CL-UD147 | FD3/M119 | *Clematis vitalba* | Friuli Venezia Giulia |
| MT629788.1 | FDp | SjForn | FD1/NA | *Spartium junceum* | Campania |
| PP196534.1 | FDp | CleTos8 | FD3/M51 | *Clematis vitalba* | Tuscany |
| PP196535.1 | FDp | DicTos20 | FD3/M51 | *Dictyophara europaea* | Tuscany |
| PP196536.1 | FDp | DicTos27 | FD3/M51* | *Dictyophara europaea* | Tuscany |
| PP196537.1 | FDp | AlnTos8 | FD1/M113 | *Alnus glutinosa* | Tuscany |
| PP196538.1 | FDp | AlnTos9 | FD2/M54* | *Alnus glutinosa* | Tuscany |
| PP196539.1 | FDp | ScaTos29 | FD1/M50 | *Scafoideus titanus* | Tuscany |
| PP196540.1 | FDp | ViTos601 | FD3/M51* | *Vitis vinifera* | Tuscany |
| PP196541.1 | FDp | ViTos997 | FD2/M54 | *Vitis vinifera* | Tuscany |
| PP196542.1 | FDp | ViTos15 | FD1/M50 | *Vitis vinifera* | Tuscany |
| AM990988.1 | BNp | PO | --- | *Hyalesthes obsoletus* | France |
