# Supplementary material for: Investigating a new alarming outbreak of flavescence dorée in Tuscany (Central Italy): molecular characterization and map gene typing elucidate the complex phytoplasma ecology in the vineyard agroecosystem
Source: Front Plant Sci. 2024 Dec 13;15:1489790. doi: 10.3389/fpls.2024.1489790 (PMC11681383; doi:10.3389/fpls.2024.1489790)
Supplement: Supplementary file 2 [file DataSheet2.pdf]

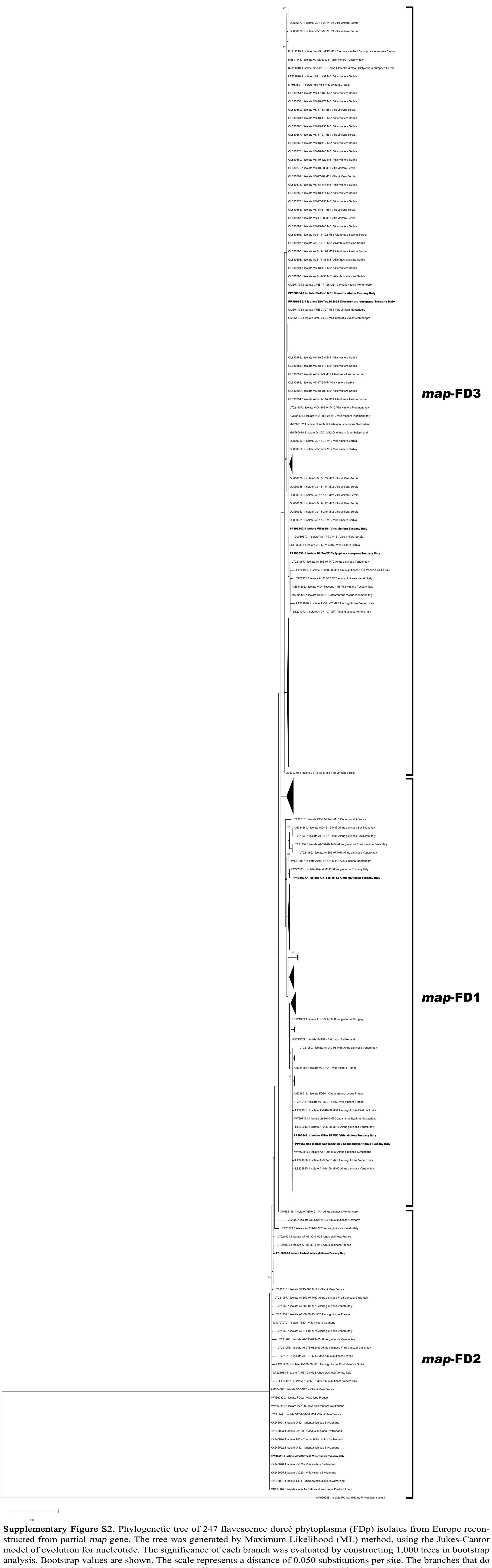

Supplementary Figure S2. Phylogenetic tree of 247 flavescence doreé phytoplasma (FDp) isolates from Europe reconstructed from partial *map* gene. The tree was generated by Maximum Likelihood (ML) method, using the Jukes-Cantor model of evolution for nucleotide. The significance of each branch was evaluated by constructing 1,000 trees in bootstrap analysis. Bootstrap values are shown. The scale represents a distance of 0.050 substitutions per site. The branches that do not contain the identified sequences have been collapsed.The isolates sequenced in this study are in bold and the subdivisions between clusters are reported on the right. For sequences from Italy, the specific region of origin is also mentioned. Bois noir phytoplasma (BNp) isolate (AM990988) was used as outgroup.
